# Radiation Dose of Abdominal and Lung Computed Tomography Based on Body Mass Index as an Indicator

**DOI:** 10.21315/mjms-04-2025-280

**Published:** 2025-08-30

**Authors:** Muhammad Kabeer Sulayman, Farouk Kabir Umar, Bashiru Lukuman, Muhammad Khalis Abdul Karim, Nor Azura Muhammad, Izdihar Kamal, Che Azura Che Abdullah, Juliana Mohd Radzi, Mazliana Ahmad Kamarudin

**Affiliations:** 1 Department of Physics, Faculty of Science, Universiti Putra Malaysia, Serdang, Selangor, Malaysia; 2 Department of Radiology, Usmanu Danfodiyo University Teaching Hospital, Sokoto, Nigeria; 3 College of Health Sciences, Universiti Malaya, Kuala Lumpur, Malaysia; 4 School of Medical Imaging, Faculty of Health Sciences, Universiti Sultan Zainal Abidin, Kuala Nerus, Terengganu, Malaysia

**Keywords:** radiation dose, computed tomography (CT), body mass index (BMI), CTDI, DLP

## Abstract

**Background:**

Radiation dose generated from computed tomography (CT) has drawn more attention to diagnostic radiology. It is a known fact that the risk of radiation-induced cancer is increasing, thereby necessitating the optimisation of dose in CT protocols. This study focused on determining the radiation dose of CT scans for the abdomen and lung using a 64-slice CT scanner to evaluate their correlation with body mass index (BMI). The objective of the study was to critically evaluate the relationship between BMI and radiation dose metrics in both CT lung and CT abdomen examinations.

**Methods:**

Data from 106 patients who underwent CT lung and CT abdomen examinations at an advanced diagnostic center were retrospectively analysed. The volume CT dose index (CTDI_vol_), dose-length product (DLP), the scan range, and skin to skin antero-posterior (AP) and lateral (LAT) of the patients were documented for further analysis. Effective dose (E) and size-specific dose estimate (SSDE) were also computed.

**Results:**

The mean BMI for CT lung was recorded as 24.85 (5.65). However, the correlation between BMI and the dose metrics (SSDE, E, DLP, and CTDI_vol_) was not significant, with correlation coefficients of 0.1278, 0.047, 0.047, and 0.1147, respectively. In contrast, the BMI for CT abdomen scans showed a moderate correlation with E (0.5898), SSDE (0.6288), DLP (0.5898), and CTDI_vol_ (0.612). The results demonstrate that BMI can be used as a radiation dose metric in the case of CT abdomen scans, but has no influence on CT lung scans.

**Conclusion:**

These results further suggest that BMI could provide radiation dose analysis, which in turn leads to optimisation of CT scan parameters.

## Introduction

In 1972, computed tomography (CT) scan was introduced into modern diagnosis, and it has become one of the most indispensable medical instruments for radiological examinations (1). High-quality CT imaging is essential to medical imaging because it benefits patients and enables accurate diagnosis. It is widely used in almost all healthcare facilities for the preliminary diagnosis of trauma. CT is superior to other imaging modalities due to its rapid image acquisition, high spatial resolution, and high contrast resolution. It is established that patients with medical implants, like ferromagnetic vascular clamps or cardiac pacemakers, remain unaffected. Consequently, the incorporation of CT into routine medical practice has significantly improved the outcome of healthcare. Notwithstanding its advantages, CT is known to contribute greater radiation exposure compared to other modalities, as reported by some studies (2). Consequently, the radiation dose from CT examinations is a crucial issue in diagnostic radiology, as the risk of radiation-induced cancer is growing, necessitating dose optimisation in CT protocols. The optimisation of CT is crucial for balancing diagnostic performance and radiation dose following the as low as reasonable achievable (ALARA) and radiation protection principles (3).

A CT scan of the abdomen provides an enhanced visualisation of abdominal structures and organs of patients compared to conventional X-rays, hence, revealing detailed information pertinent to injuries or disorders present in the abdominal organs. During a CT scan of the abdomen, it is imperative to take into consideration radiation doses and size-specific dose estimations (4). CT scans are widely employed in medical imaging due to their ability to provide accurate anatomical information. Patients are exposed to ionising radiation during CT examination, which carries significant hazards that include the potential risk of cancer. Therefore, the optimisation of CT scanning protocols and the accurate precision of radiation dose are crucial to minimising patient exposure. Assessing the radiation dose received by each patient during a CT examination is known for its intrinsic challenges. Interest in this area is rapidly increasing, primarily due to the increase in the use of CT scans and global concern about the potential risks linked to radiation exposure from medical radiation. The CT dose report gives the radiation dose output of the scanning machine after scanning a given patient. It is represented as the volume CT dose index (CTDI_vol_) and dose-length product (DLP).

The CT lung examination is used in medical diagnosis to evaluate all the organs found within the thoracic region, encompassing the lungs, the heart, the bones, and the blood vessels. It provides well-detailed cross-sectional images that assist in diagnosing diseases such as aortic and thoracic tumours, pulmonary embolism, and other cardiovascular disorders. It is important in both emergency and routine clinical investigations as it can detect even minor ailments. The radiation doses of CT lung and CT abdomen are different because of the differences in the structures of their anatomy and the purpose of the scan. The CT of the lung needs a lower radiation dose than CT abdomen because lung tissues are denser and provide a natural contrast between surrounding tissues and air-filled space. Conversely, CT of the abdomen involves imaging denser organs such as the kidneys, the intestines, and the liver. These organs need much higher radiation doses to achieve good image quality and precise diagnosis. The CT lung scan has an effective dose (E) ranging from 1.5 to 7.0 mSv, whereas that of the CT abdomen scan ranges from 5.0 to 25.0 mSv, contingent upon the scanning protocol used and patient characteristics. These differences show the importance of dose optimisation techniques for different types of CT scans to ensure the patient’s safety while achieving diagnostic efficacy.

The personalised radiation dose for a patient is not quantified by these criteria, as it is influenced by the size of the patient. The size-specific dose estimate (SSDE) is a dose metric that accounts for the size of the patient in the dose calculation. The American Association of Physicists in Medicine (AAPM) endorsed and advocated for the utilisation of this metric in the reporting of the radiation dose to patients in CT. These metrics have increasingly gained more utilisation and acceptance over time. The SSDE is determined by multiplying the CTDI_vol_ by a conversion factor that is derived from the physical measurement of the antero-posterior (AP) and lateral (LAT) skin-to-skin patient diameters at the midline on CT localiser images (5). The aim of this study is to evaluate and assess the viability of using body mass index (BMI) as a replacement for body diameters in determining patient effective diameter (D_EFF_). Therefore, the assessment of SSDE during CT scanning would be more user-friendly, making a favourable contribution to optimising patient radiation dose.

## Methods

### Patient Criteria

This was a retrospective cross-sectional study conducted using anonymised patient data from routine CT abdomen and CT lung scans in state of Sokoto State Advanced Medical Diagnostic Center, Nigeria. Data were stratified based on BMI categories to evaluate the association between BMI and CT dose metrics. This study received approval from the research ethics committee, and consent was not required from the patients. Data were collected from June 2021 to June 2023. The study included 76 adult patients who underwent a routine abdominal CT scan and 30 patients who underwent a CT lung scan. This study included adult patients (≥ 18 years old) who underwent routine CT abdomen or CT lung examinations during the study period, with available demographic data and complete dose reports, including CTDI_vol_, DLP, and scan parameters. Exclusion criteria included patients with insufficient scan data, missing BMI statistics, or those who had nonstandard CT protocols such as contrast-enhanced phases or interventional procedures. This was a retrospective investigation; thus, all eligible patients from the study period were included without a sample size calculation. The sample size was found adequate to detect overall trends in dosage parameters across BMI groups.

### Scan Protocol

The data were obtained from the picture archiving and communication system (PACS) of 16 multi-slice CT scan equipment (CT Revolution, GE HealthCare, Chicago, IL, US). While rotating, the scanner is capable of reconstructing 16 slices, each of which has a thickness of 1.25 mm. This scanner is one of the most sophisticated in Nigeria. It generates high-resolution 3D images that outperform those produced by conventional single-slice CT scans. The tube voltage is set at 120 kVp. Table 1 shows that the tube current is automatically adjusted between 50 mA and 250 mA based on the patient’s size, attenuation characteristics of the scanned area, and body composition. The machine employs an automated exposure control (AEC) to improve image quality while reducing radiation dose, dynamically adjusting the current according to the patient’s anatomical and density variations.

The CTDI_vol_ is the quantitative evaluation of the radiation dose associated with a scanning protocol. Where CTDI_w_ is the weighted or average CTDI given across the field of view and pitch is the ratio of the table feed (in mm) per 360° gantry rotation to the nominal collimated beam width. It entails the distribution of radiation dose resulting from the consecutive rotations of the X-ray source, taking into consideration any gaps or overlaps. The equation which represents its mathematical expression is as follows:


(1) $$CTDI_{vo\text{l}}=\frac{CTDI_{w}}{pitch}$$

The DLP, which is obtained from the console, represents the total dose absorbed by a patient throughout the scanning period. It is quantified in milligray-centimetres (mGy. cm), and it is very important for assessing scan doses when the scan lengths are uniform. The DLP can be mathematically derived using the equation as follows:


(2) $$DLP=CTDI_{vol}\times ScanLength$$

The values for the DLP, the CTDI_vol_ values, and the scan range for every scanned patient were recorded from each CT dose report obtained from the console.

The E was calculated by multiplying the DLP by the conversion coefficient factor, *k*. The equation is:


(3) E=DLP×k,

where *k* is 0.0124 μSv/mGy.cm as obtained from International Commission on Radiological Protection Publication (5).

### BMI Measurement

Prior to the CT scan, weight and height measurements were taken for each patient, and their BMI was computed. The patient groups were categorised based on BMI data, with underweight defined as a BMI less than 18.5 kg/m^2^, normal weight defined as a BMI between 18.5 and less than 25.0 kg/m^2^, overweight defined as a BMI between 25.0 and less than 30.0 kg/m^2^, and obese defined as a BMI equal to or greater than 30.0 kg/m^2^.

### Body Diameter Measurement

**Figure 1:**
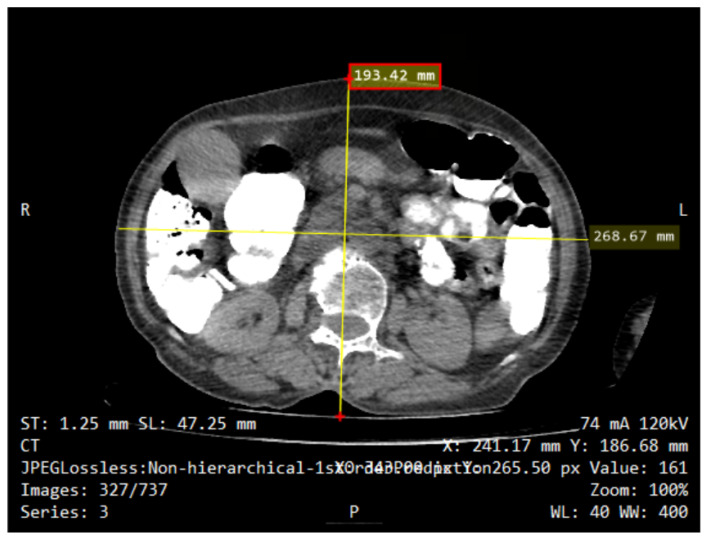
Diameter measurement of LAT/AP for calculating D_EFF_

The images were assessed using the PACS in a Digital Imaging and Communications in Medicine (DICOM) format. According to the standards outlined in the AAPM Report 204 with the tittle Size-Specific Dose Estimates (SSDE) in Pediatric and Adult Body CT Examinations, body diameters were measured at the midline level, specifically on the CT localiser images. The diameter measurements were manually done using the electronic calliper found in the PACS system. Each CT scan image employed a consistent window level and setting. The AP and LAT diameters were measured in centimetres (cm) on the localiser images to determine the maximum skin-to-skin dimensions. The AP refers to the measurement of the skin-to-skin diameter from front-to-back on the side view image at the centre level. On the other hand, LAT is the measurement of the skin-to-skin diameter at the centre level from side to side on the front-to-back view image. The diameter of the circle with the same area as the patient’s cross-sectional area at a particular z-axis level (more precisely, the midline level) is known as the effective diameter (D_EFF_) (Figure 1). It can be computed using the following equation, which shows the geometric mean of LAT and AP:


(4) $$D_{EFF}=\sqrt{AP\times LAT}$$

Thus, the calculation of SSDE can be deduced from the following equation:


(5) $$SSDE=CTDI_{vol}\times f$$

The value of *f*, which solely depends on the value of D_EFF_, can be determined from Table 1D of the AAPM Report 204 (5).

### Statistical Analysis

Data were initially organised using Microsoft Excel 2010 (Microsoft, Redmond, WA, US). Statistical analyses were conducted using Statistical Package for the Social Sciences (SPSS) version 25 (IBM Corp., Armonk, NY, US). Descriptive statistics, such as mean, range, and standard deviation (SD), were computed. Pearson correlation coefficients were calculated to assess the relationships between dose indices, BMI, and body diameters. Linear regression models were used to evaluate the impact of dose metrics (CTDI_vol_, DLP, SSDE, and E) on BMI. A *P*-value below 0.05 was considered statistically significant.

## Results

### Demography of the Patients

The study comprised 106 patients (60 females and 46 males), 30 for CT lung and 76 for CT abdomen. The mean (SD) of age is 51.47 (18.75) for CT lung and 50.62 (16.64) for CT abdomen, ranging from 18 to 89 years. The mean demographic characteristics of patients who had CT lung and CT abdominal examinations are shown in Table 2. The table includes data for the total number of patients, age, weight, height, BMI, the AP/LAT diameter measurements, and the D_EFF_. The sample size for CT lung (30) is smaller than that for CT abdomen (76), which may show a significant difference in the statistical analysis. They both have a similar mean age, which implies that the patients undergoing lung and abdomen CT scans are of comparable age, though the patients for CT lung show a slightly wider age range of 51.47 (8.75). The mean weight of patients who underwent CT abdomen is 70.43 kg with an SD of 18.25 kg, which is higher than that of patients who underwent CT lung scans. The mean height of the patients is very similar in both cases, with a slight variability in CT abdomen. Patients who underwent CT abdomen have a higher mean BMI of 27.37 (6.27), indicating a higher average body mass relative to height than those who underwent CT lung. The CT abdomen has a higher mean of 209.93 (30.53) for the AP midline measurement than that of the CT lung. This suggests differences in body composition during the scans. Also, there is a higher mean LAT dimension of 271.32 with an SD of 33.04 for CT abdomen than that of patients who underwent the CT lung scan, with less variability (lower SD). The D_EFF_ values for both groups are similar, though the CT abdomen patients have a slightly higher mean and lower variability of 21.89 (1.38). The demographic data show that while the patients who underwent CT lung and CT abdomen are similar in height and age, notable differences can be seen in weight, BMI, and body dimensions, which could influence the scan protocols and diagnostic outcome.

Table 3 presents the comparison of different dose metrics (CTDI_vol_, DLP, SSDE, and E) for patients who are undergoing CT lung and CT abdomen across various BMI categories. The tube voltage remains constant at 120 kVp for all BMI categories, which signifies a common practice in CT protocol. However, the tube current-time product (mAs) increases progressively with BMI, reflecting the need for higher exposure in patients with larger body habitus. Both lung and abdominal CT images show an increase in mAs when BMI increases. This is predictable given the greater mAs required to enter individuals with bigger body habitus. All dose measures, including CTDI_vol_, DLP, SSDE, and E, indicate an increasing trend as BMI increases. The increasing trend of dose metric with BMI is consistent with the findings from a previous study, which explains that CT radiation dose increases as the patient size increases (6).

The higher dose metrics that can be noticed for abdominal CT compared to CT lung align with the study by Saltybaeva et al. (7), which reported higher radiation doses for CT abdomen compared to CT lung across different BMI categories. The SSDE values recorded in this study range from 5.0 to 14.0 mGy. These figures are in line with the range reported by Binta et al. (8), where it was stated that SSDE values for CT abdominal scans fall within the range of 4 to 25 mGy. The E values in this study vary from 1.5 (0.03) to 1.7 (0.20) mSv across all BMI categories, as in the case of CT lung, and from 2.4 (0.42) to 6.5 (2.00) mSv for CT abdomen. It is noted that these values are slightly lower than those indicated in previous studies, including the review by Mettler et al. (9), in which they reported a typical E value of 7 mSv for a CT lung scan and 8 mSv for a CT abdomen. The values for DLP in this study correspond with earlier research, such as the study by Tsalafoutas and Koukourakis (10), where the values of DLP were documented to be between 330 and 460 mGy·cm for CT lung and 460 to 640 mGy·cm for CT abdomen.

Table 4 summarises the total dosage metric used in this study. The average CTDI_vol_ for CT lung is 4.56 mGy (1.43), while for CT abdomen it is 6.50 mGy (3.00). These findings are congruent with the observed pattern in the categorised BMI data, which revealed that CT abdomen had higher doses than CT lung. The results are lower than those previously reported in certain literature (11), with recorded CTDI_vol_ values of 4.56 mGy for the CT lung and 6.5 mGy for the CT abdomen. The lower values in this study could be associated with dose reduction techniques.

The mean value for all the DLP in this study for CT lung is 107.37 (5.86), and for CT abdomen is 315.6 (152.50). The DLP for CT abdomen falls within the range of the categorised BMI data. Despite exhibiting a slightly high SD, this indicates the variability among all BMI categories. The DLP mean value for CT lung aligns with the previous study. However, the DLP mean value for CT abdomen is slightly lower than reported values. This can be noticed in the study by Atlı et al. (12), where they reported DLP values of 195 for chest CT and 477 for abdominal CT. The SSDE for all patients in this study recorded a mean value of 7.69 (2.25) for CT lungs and 10.70 (4.50) for CT abdomen. These figures align with those reported in the study by Brat et al. (13), in which a mean SSDE value of 7.3 mGy was recorded for CT lung and 13.9 mGy for CT abdomen. The mean values of E for all the patients who underwent CT lung in the present study are 1.61 (0.09), whereas those for the abdomen are 4.70 (2.30).

In this study, the E is marginally lower than those documented in some past literature, particularly for CT abdominal examination, which documented an E of 6.9 (2.9) for CT lung and 8.9 (2.8) for CT abdomen (3). The recorded lower value in the present research may indicate an effective technique of dose reduction. Nonetheless, the absolute dose values for CTDI_vol_, DLP, and E are marginally lower than those documented in some studies. This could be a result of the implementation of the E reduction technique employed by the centre where the study was carried out. The techniques include automated tube current modulation (ATCM), iterative reconstruction (IR), or low kilovolt (kV) settings. The elevated SD, particularly for the CT abdomen, show the variability among various BMIs, which is an important factor in CT dose optimisation for BMI.

**Figure 2:**
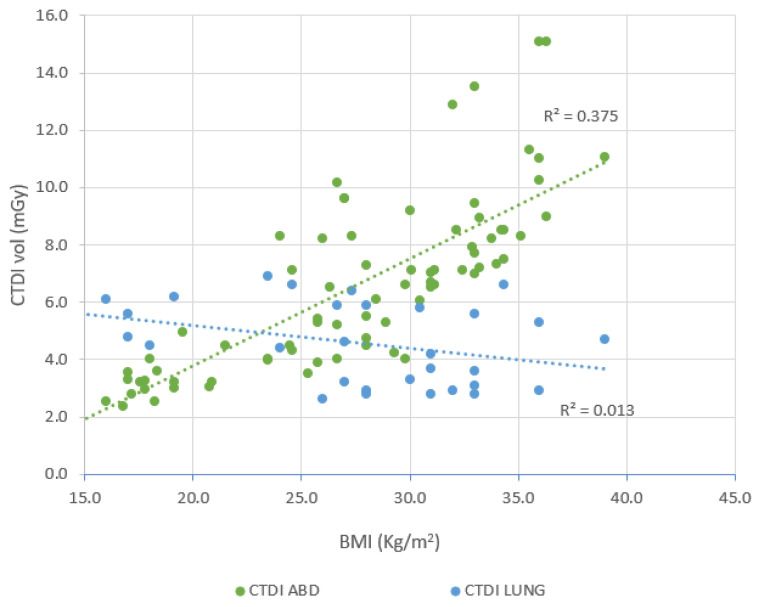
Correlation between CTDI_vol_ and BMI *r* **=** 0.612 for CT abdomen; a negative correlation of 0.1147 for CT lung

A multiple linear regression analysis was performed to determine the effect of CTDI_vol_, DLP, E, and SSDE on BMI. A clear positive correlation between CT abdomen and BMI is shown in Figure 2. As the BMI rises, the CTDI_vol_ value increases as well. The value of correlation is 0.612, indicating a relatively strong correlation between BMI and CTDI_vol_ in the case of CT abdomen. A comparable trend is seen in past research (14, 15), in which a strong correlation of 0.8 was recorded between CTDI_vol_ and BMI. The corresponding R^2^ value of 0.375 suggests that 37.5% of the variation in CTDI_vol_ for CT abdomen can be attributed to differences in BMI. In contrast, CT lung scans show a weak negative correlation (*r* = 0.1147), implying that the relationship between BMI and CTDI_vol_ is more specific and significant in the abdominal region.

**Figure 3:**
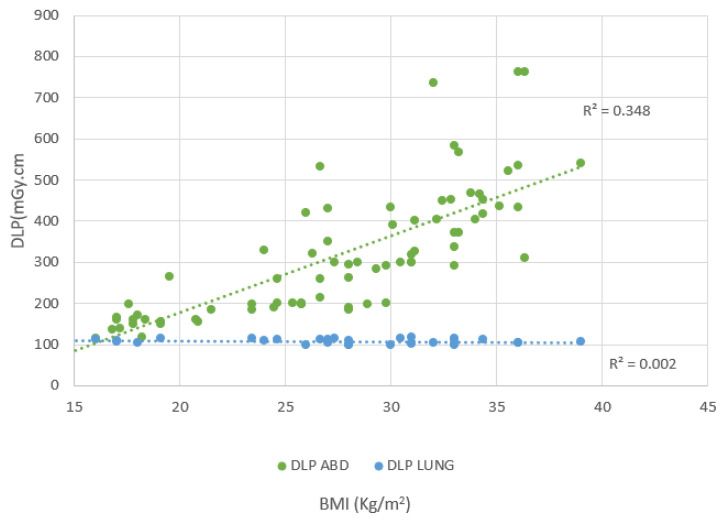
Correlation between DLP and BMI Positive correlation with *r* = 0.5898 for CT abdomen; poor correlation for CT lung

There is a positive correlation between BMI and DLP, as indicated by the trend line with a positive slope, which shows that DLP for the CT abdomen increases with increasing BMI, demonstrating a moderate linear relationship as shown in Figure 3. The *r* value of 0.589 shows a moderate correlation. This is in line with previous studies (15, 16). There is approximately no correlation between BMI and DLP for the CT lung, as indicated by the nearly horizontal trend line. The correlation coefficient (*r* = 0.0471) reflects a very weak relationship. This suggests that only 0.22% of the variability in DLP for CT lung can be explained by BMI (*R*^2^ = 0.0022), indicating that BMI has virtually no effect on DLP in lung CT scans.

**Figure 4:**
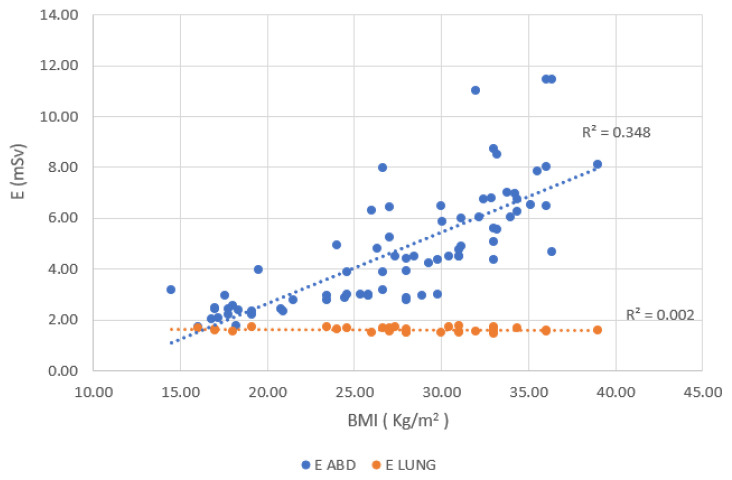
Correlation between E and BMI Positive correlation for CT abdomen with r = 0.589; relatively no correlation for CT lung

The correlation between BMI and E for CT lung and CT abdomen was demonstrated in Figure 4. A strong positive connection of 0.5898 can be observed between BMI and E for the CT abdomen, while a weak correlation of 0.0471 can be observed for CT lung. This trend is similar to a previous study (2), which shows a positive correlation of 0.61 between BMI and E variables. This explains that patients with a higher BMI require a higher dose due to the increased thickness of their body tissues. For CT lung, the findings align with the study by Heston and Jiang (17), who reported relatively no influence of BMI on CT lung.

**Figure 5:**
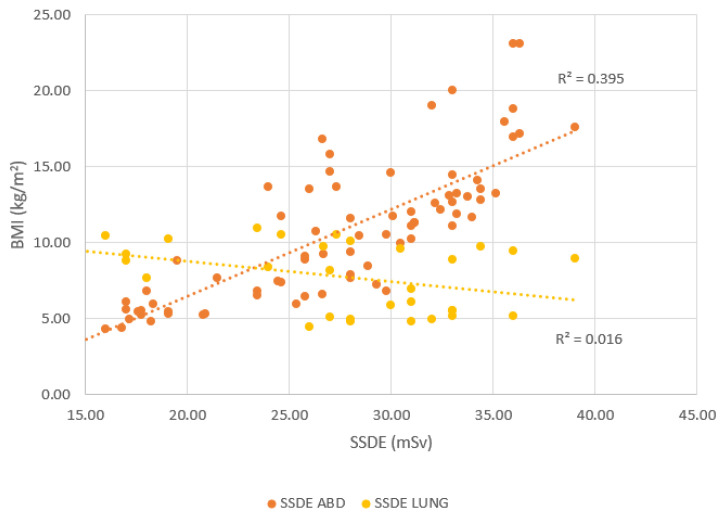
Correlation between SSDE and BMI Good correlation for CT abdomen with *r* = 0.6288; poor correlation for CT lung with *r* = 0.1278

The correlation between SSDE and BMI for CT abdomen and CT lung is shown in Figure 5. The SSDE for CT abdomen shows a positive correlation of 0.6288 with BMI. This aligns with findings in a previous study (16), where a strong correlation of 0.85 was reported between SSDE and BMI, signifying that SSDE increases with patient size for CT abdomen. They reported that SSDE gives a more accurate radiation dose estimation than CTDI_vol_ alone, especially when patients have different sizes. For CT lung, there is a weak negative correlation of 0.1278 between SSDE and BMI, which suggests that BMI has little influence on SSDE for CT lung. This was reported in a similar study by Binta et al. (8), who found that SSDE for CT lung was almost constant across different BMIs, in contrast to CT abdomen, where SSDE increased with size.

**Figure 6:**
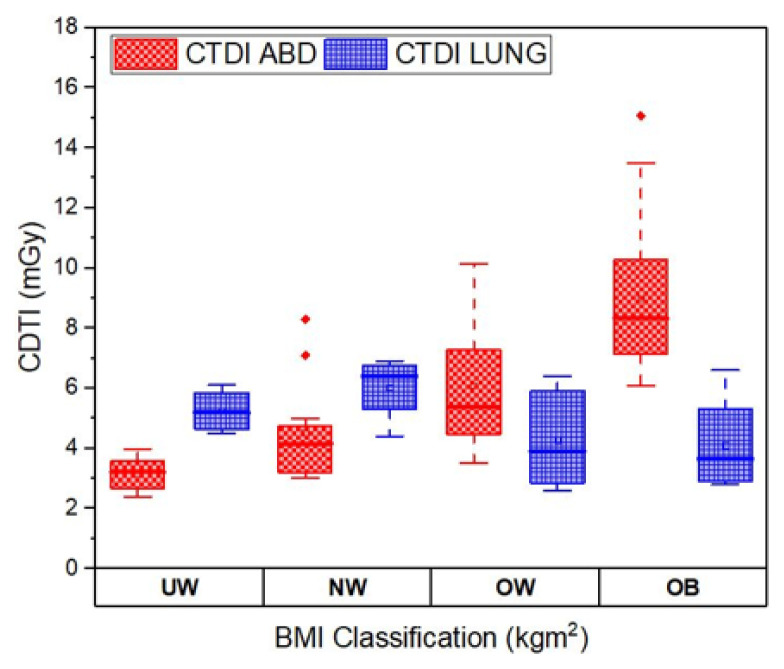
Box plot comparison between CTDI_vol_ for CT abdomen and CT lung UW = underweight; NW = normal weight; OW = overweight; OB = obesity

The box plot for CTDI_vol_ values of CT abdomen and CT lung for different BMI classification groups is shown in Figure 6. The box plot compares the CTDI values across the different BMI groups. This is essential in understanding the influence of patient size on radiation dose during CT examinations. Previous studies have indicated that the size and body habitus of the patient influence the CTDI_vol_ significantly during a CT abdominal exam. Patients with large BMI in the obese and overweight category need a higher value of CTDI_vol_ to attain better and clearer image quality. The box plot illustrates this trend, showing that CTDI_vol_ values for CT abdomen increase from the underweight to the obese categories. The impact of BMI on CTDI_vol_ for CT lungs is not clearly evident, as can be seen in the box plot.

**Figure 7:**
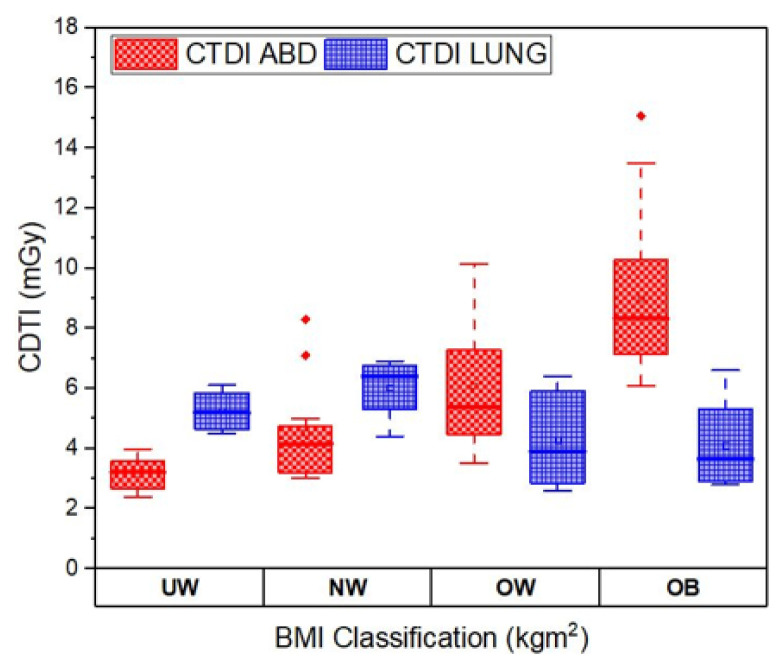
Box plot comparison between DLP for CT abdomen and CT lung UW = underweight; NW = normal weight; OW = overweight; OB = obesity

The comparison of the DLP values for CT abdomen and CT lung across different BMI categories is shown in Figure 7. The box plot shows DLP for CT abdomen to significantly increase as BMI increases, peaking in the obese category. At the same time, the DLP for CT lung is relatively constant across all BMI categories. This finding is in line with the study by Lee et al. (18), in which higher BMI is associated with higher DLP in the case of CT abdomen due to increased tissue density. On the contrary, DLP for CT lung shows minimal variation, implying that BMI has less influence on CT lung. The increase recorded in the DLP of CT abdomen with BMI underscores the need to adjust the protocols for dose optimisation in patients with higher BMI.

**Figure 8:**
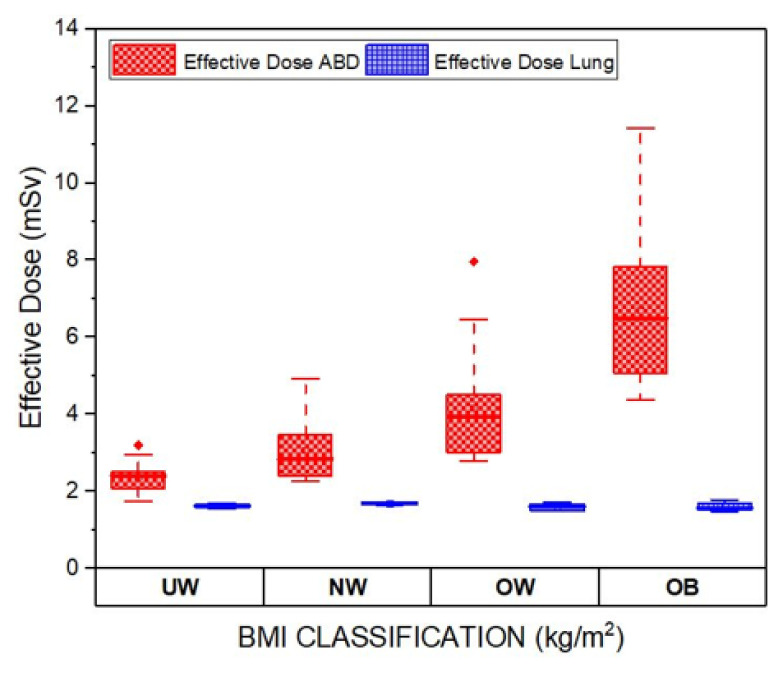
Box plot comparison between E for CT abdomen and CT lung UW = underweight; NW = normal weight; OW = overweight; OB = obesity

The box plot comparing the E for CT abdomen and CT lung across different BMI categories is shown in Figure 8. For CT abdomen, the box plot explains that the higher the BMI, the higher the E. This shows that denser tissues require greater radiation penetration. Such a trend has been reported by Dolenc et al. (19). The box plot for CT lung shows consistency across different BMI categories, similar to the findings by Sebelego et al. (20), where they reported that BMI has minimal influence on CT lung.

**Figure 9:**
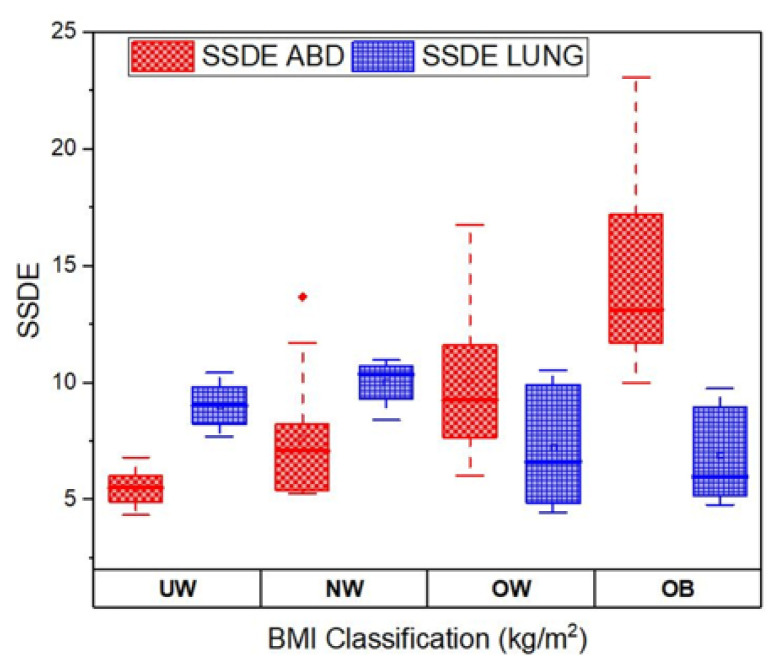
Box plot comparison between SSDE for CT abdomen and CT lung UW = underweight; NW = normal weight; OW = overweight; OB = obesity

In the comparison between SSDE of CT lung and CT abdomen across all BMI categories, there is a trend of increasing SSDE as BMI increases for CT abdomen examination, as shown in Figure 9. This observation can also be noticed in a past study, which reported that higher radiation doses are required for patients with large BMI during CT abdomen scans to achieve optimal image quality (21). This is due to the increased tissue and adipose material in such patients. As a result, more radiation is needed to penetrate these tissues effectively. The difference in SSDE values between CT lung and CT abdomen is shown in Figure 8. Across all BMI categories, the SSDE values for CT abdomen are generally higher than the SSDE values for CT lung. This difference can be linked to the varying tissue densities and attenuation properties that exist between them. The abdomen has denser tissues compared to the lungs.

## Discussion

This study aimed to investigate the relationship between BMI and radiation dose measurements in CT lung and CT abdomen examinations. The data indicated a clear distinction between the two anatomical areas (abdomen and lung); BMI showed no significant relationship with dose metrics in CT lung scans, but it did exhibit a moderate and statistically significant correlation in CT abdomen scans. These findings give new insights into how patient size, as expressed by BMI, affects radiation exposure in different CT protocols. The moderate correlations observed between BMI and dose indices in CT abdomen align with findings from Brix et al. (22) and Zarb et al. (23), who reported that larger patient body size contributes to increased ATCM, resulting in higher CTDI_vol_ and DLP values. This is consistent with the use of dose modulation systems that adjust mA based on patient attenuation, which is more pronounced in abdominal regions due to soft tissue density and variable girth.

On the other hand, the lack of a significant relationship between BMI and dose parameters in CT lung scans supports the observations by Lin et al. (24) who suggested that the relatively uniform thoracic anatomy and lower attenuation characteristics of lung tissue may reduce the variability in dose requirements, even in patients with higher BMI. In their findings, the signal-to- noise ratio (SNR) and contrast-to-noise ratio (CNR) demonstrated only weak correlations with body composition indicators, including BMI and body surface area (BSA). This suggests that dose modulation in the chest is less sensitive to BMI-related variations in patient habitus compared to the abdominal region.

Additionally, the predictive ability of BMI for radiation dose in abdominal CT demonstrated by this research is in agreement with a previous study (25), which emphasised the role of patient-specific factors in dose optimisation. This supports the idea that BMI, as a proxy for patient size, might be a valuable signal for protocol customisation, especially in abdominal imaging (26).

However, it is crucial to remember that BMI alone may not accurately reflect patient geometry or composition. Prior research suggested using the SSDE as a more reliable tool for calculating patient radiation dose in myocardial perfusion imaging with single-photon emission computed tomography (MPI SPECT/CT) scans (27). Although this study used BMI since it is readily available in typical clinical settings, future research may benefit from merging BMI with cross-sectional diameter data to boost the precision of dosage prediction models.

The CTDI and DLP of CT abdomen and CT lungs have different correlations with BMI owing to variations in tissue density, X-ray attenuation properties, and image acquisition protocols. A primary factor that is influencing this correlation is X-ray attenuation and tissue composition. The abdominal region contains many organs, which are all known to have varying levels of radiation sensitivity, including the liver, kidneys, stomach, and intestines. Therefore, dose optimisation is crucial in abdominal CT to minimise the potential radiation risks to these radiosensitive structures. These are all known to significantly influence X-ray attenuation. Large patients with higher BMI are generally associated with increased levels of subcutaneous and visceral fat that absorb greater amounts of radiation. To offset this, the CT scanners are designed to increase the tube current (mA), leading to increased CTDI and DLP values. Conversely, CT lung imaging involves air-filled regions that have significantly lower attenuation as compared to abdominal tissues. Even in larger patients with high BMI, the lung retains its low-density structure, indicating that the necessary radiation dose does not significantly increase as it does in abdominal CT. Another factor that is significant is the operation of AEC. In abdominal CT, the AEC systems modulate the tube current according to the body dimensions of the patient to achieve adequate image quality. As BMI rises, the tube current is increased by AEC to compensate for the increased attenuation caused by surplus fat and soft tissue, thereby resulting in a stronger correlation between the radiation dose metrics (CTDI and DLP) and the BMI. However, in CT lung imaging, AEC does not necessarily require significant adjustment due to the inherently low attenuation of lung tissue. As a result, even in obese patients, the increase in radiation dose is minimal compared to what is observed in abdominal imaging.

## Conclusion

Optimising the dose is crucial in modern radiology, especially in the case of CT scans, where it is crucial to find the right balance between radiation dose and image quality. To be efficient and valuable, any available technique that can be helpful in the calculation and optimisation of the radiation dose received by a patient should be easy to use and have the ability to replicate correctly. The current study demonstrates that the BMI of a patient can be utilised to estimate the radiation dose in the CT abdomen precisely and may not affect the CT lung, eliminating the necessity of measuring LAT and AP diameters for calculating SSDE during CT scans. In summary, CTDI and DLP correlate strongly with BMI for CT abdomen due to the higher attenuation from soft tissue and fat, requiring increased tube current. In contrast, for CT lung, the correlation is weaker or absent because lung tissue has low attenuation, and AEC does not significantly adjust the dose based on BMI. Nevertheless, the dose values, mainly for E, show a lower end of the reported ranges. This could be due to the use of dose optimisation techniques in the present study.

## Figures and Tables

**Table 1:** Scanning acquisition parameter

Protocol	Parameter
Tube voltage (kVp)	120
Pitch	1.75
Orientation	Caudocranial
Scanning mode	Helical
Slice thickness	1.25
Contrast media	No

**Table 2:** The mean value of patient demographics

	CT lung	CT abdomen
*n*	30	76
Age	51.47 ± 18.75	50.62 ± 16.64
Weight (kg)	66.33 ± 17.76	70.43 ± 18.25
Height (m)	1.62 ± 0.06	1.61 ± 0.08
BMI (kg/m^2^)	24.85 ± 5.65	27.37 ± 6.27
AP	197.52 ± 33.64	209.93 ± 30.53
LAT	258.07 ± 40.27	271.32 ± 33.04
D_EFF_	21.28 ± 1.64	21.89 ± 1.38

**Table 3:** Mean and SD of dose metrics based on BMI for CT lung and CT abdomen

	Underweight		Normal weight	

	Lung	Abdomen	Lung	Abdomen
*n*	7	12	5	13
kV	120	120	120	120
mAs	52.3 ± 2.3	64.9 ± 13.2	53.8 ± 1.9	77.3 ± 12.7
CTDI_vol_	2.8 ± 0.2	3.2 ± 0.5	3.5 ± 0.5	4.4 ± 1.6
DLP	100.4 ± 1.7	157.0 ± 28.7	105.4 ± 7.3	202.5 ± 52.5
SSDE	5.0 ± 0.30	5.5 ± 0.84	5.8 ± 0.80	7.5 ± 2.60
E	1.5 ± 0.03	2.4 ± 0.42	1.6 ± 0.10	3.0 ± 0.81

	**Overweight**		**Obesity**	

	**Lung**	**Abdomen**	**Lung**	**Abdomen**

*n*	11	18	11	18
kV	120	120	120	120
mAs	57.6 ± 5.5	90.9 ± 23.0	57.6 ± 5.5	90.9 ± 23.0
CTDIvol	5.1 ± 0.8	6.3 ± 2.0	5.1 ± 0.8	6.3 ± 2.0
DLP	108.8 ± 3.3	285.2 ± 97.6	108.8 ± 3.3	285.2 ± 97.6
SSDE	8.6 ± 1.20	10.5 ± 3.20	8.6 ± 1.20	10.5 ± 3.20
E	1.6 ± 0.40	4.3 ± 1.52	1.6 ± 0.40	4.3 ± 1.52

**Table 4:** Mean value of dose metric for CT lung and CT abdomen

	Lung	Abdomen
*n*	30	76
CTDI_vol_	4.56 ± 1.43	6.50 ± 3.00
DLP	107.37 ± 5.86	315.60 ± 152.50
SSDE	7.69 ± 2.25	10.70 ± 4.50
E	1.61 ± 0.09	4.70 ± 2.30
